# SCADA-Based Message Generator for Multi-Vendor Smart Grids: Distributed Integration and Verification of TASE.2 †

**DOI:** 10.3390/s21206793

**Published:** 2021-10-13

**Authors:** Petr Ilgner, Petr Cika, Martin Stusek

**Affiliations:** Department of Telecommunications, Brno University of Technology, 616 00 Brno, Czech Republic; cika@vut.cz (P.C.); xstuse01@vut.cz (M.S.)

**Keywords:** IEC 60870-6, TASE.2, smart grid, traffic generator, Apache JMeter, Apache Kafka, SCADA, MongoDB, Microsoft Azure

## Abstract

Recent developments in massive machine-type communication (mMTC) scenarios have given rise to never-seen requirements, which triggered the Industry 4.0 revolution. The new scenarios bring even more pressure to comply with the reliability and communication security and enable flawless functionality of the critical infrastructure, e.g., smart grid infrastructure. We discuss typical network grid architecture, communication strategies, and methods for building scalable and high-speed data processing and storage platform. This paper focuses on the data transmissions using the sets of standards IEC 60870-6 (ICCP/TASE.2). The main goal is to introduce the TASE.2 traffic generator and the data collection back-end with the implemented load balancing functionality to understand the limits of current protocols used in the smart grids. To this end, the assessment framework enabling generating and collecting TASE.2 communication with long-term data storage providing high availability and load balancing capabilities was developed. The designed proof-of-concept supports complete cryptographic security and allows users to perform the complex testing and verification of the TASE.2 network nodes configuration. Implemented components were tested in a cloud-based Microsoft Azure environment in four geographically separated locations. The findings from the testing indicate the high performance and scalability of the proposed platform, allowing the proposed generator to be also used for high-speed load testing purposes. The load-balancing performance shows the CPU usage of the load-balancer below 15% while processing 5000 messages per second. This makes it possible to achieve up to a 7-fold improvement of performance resulting in processing up to 35,000 messages per second.

## 1. Introduction

The fourth industrial revolution (Industry 4.0) started to raise new challenges for communication between devices, especially in industrial environments where reliability and performance are the key features. From the communication technologies point of view, the standardization bodies created an entirely new group of representatives designed from scratch to cover the never-seen requirements. Even though technologies for wired or wireless data transmission in harsh conditions were standardized, e.g., Narrowband IoT or LTE Cat-M for the industry-driven wireless data communication, the already deployed systems for the industry communication, i.e., supervisory control and data acquisition (SCADA) systems, are still in operation. As the industrial sector has to play save and ensure reliable and secure communication, mainly for the critical infrastructure, one can not expect the seamless integration of the new communication technologies [1].

But it is not only about the technologies. For example, in the case of massive machine-type communication (mMTC), the communication (application) protocols started to come to light and play, at the very least, an equally important role. For instance, the Modbus protocol is a perfect example as it represents the legacy representative for communication of programmable logic controller (PLC) communications. It has been replaced by the Profinet bus, but it still took more than two decades to migrate and reach the expected communication parameters [1].

Within the mMTC, the areas having the most significant momentum in the course of the past two years are remote meetering and power automatization. Both stay for the typical representatives of the SCADA systems as they are primarily used to monitor and control the parts of the critical infrastructure, e.g., (i) smart electricity metering and management, (ii) system health checks, and (iii) security reports, as well as (iv) periodic system status reporting. The importance is highlighted even more as renewable energy sources (wind and solar power plants) are preferred over of legacy energy sources, e.g., coal power plants. Then the national supervisory authorities can monitor the system status of subordinate control centers and exchange data among them [2].

The insights mentioned above are further confirmed by the prediction from the leading industry companies. Even though the forecast of 50 billion devices by 2022 may be exaggerated, the general trend that early analysts predicted is indisputable. Current numbers vary from 6 to 9 billion devices, whereas forecasts predict 20 to 30 billion IoT devices around 2022. In addition, Gartner launched a forecast suggesting that industrial Internet of things (IIoT) devices will represent around 37% of the global volume, accounting for about 57% of the overall IoT expenditures in 2020. This trend includes sending the acquired data to various on-premises or cloud-based management services, providing long-term storage of the received data, and further processing information to visualize system status, minimize failures or increase future efficiency.

This paper focuses specifically on the data transmissions using the sets of standards IEC 60870-6 (ICCP/TASE.2). The traffic generator for the TASE.2 protocol is designed together with the data collection back-end, where load balancing functionality is implemented. Although this may not be noticeable at first glance, the industrial protocols’ performance analysis in smart grid networks is difficult to obtain. Even more, the performance of the TASE.2 protocol generator is not possible to find, to the best of the authors’ knowledge. Therefore, the research goals lead us to the testing scenarios, where the performance and level of availability of the load balancers deployed in the smart grid infrastructure will be tested. The intended implementation opens the door for the high-availability together with the load-balancing—enabling the network redundancy and the scalability of the complete communication platform.

### Main Contribution

In terms of SCADA systems, high reliability and communication security are the most crucial parameters which all parts of the system must ensure. To this aim, we present a newly developed framework, which allows us to assess and even improve the performance, security, and reliability of the TASE.2 protocol for use in SCADA systems.

The TASE.2 protocol is already widely used to exchange data between control centers and power plants in the distribution power grid [3,4]. Hence, in this paper, we propose a newly designed TASE.2 data generator that can be used for conformity, performance, or various attacks vulnerability testings. The developed application is capable of generating real TASE.2 traffic for both PUSH and PULL scenarios. It is also capable of simulating multiple client/server nodes on a single machine.

Further, we propose utilizing the Apache Kafka event streaming platform in combination with load balancer and collector servers to ensure high availability, ubiquitous data access, and traffic load-balancing [5,6]. The data traffic can be evenly distributed over multiple brokers and stored in a geographically separated database using the Apache Kafka broker, ensuring high data availability. On top of that, utilization of load balancer servers allows to arbitrary scale the overall system performance on-demand. Aside from the load-balancing, the proposed scheme also allows increasing reliability in any case of a server failure when the traffic is automatically dispersed among the remaining nodes.

The rest of this paper is organized as follows. In Section 2, the protocol in question (TASE.2) is described in detail. Further, in Section 3, the developed TASE.2 traffic generator and the data collection backend with the implemented load balancing functionality are described. The thorough performance evaluation of the designed generator is introduced Section 4. Finally, the conclusions are drawn in Section 5.

## 2. Technology Background

For the purpose of this work, we used TASE.2 protocol defined in the fifth part of the IEC 60870 standard set. This protocol is designed to enable the exchange of time-critical information among transmission system operators (TSO)s via conventional wide area networks (WAN)s or local area networks (LAN)s [7]. The protocol consists of three main parts, defined by the International Electrotechnical Commission (IEC) Technical Committee (TC) 57, which describes functional and object models. Namely, it covers TASE.2 (i) services and protocols (IEC 60870-6-503) [8], (ii) functional profile for application service in end systems (IEC 60870-6-702) [9], and (iii) object models (IEC 60870-6-802) [7,10].

### 2.1. TASE.2 Architecture

**Figure 1:**
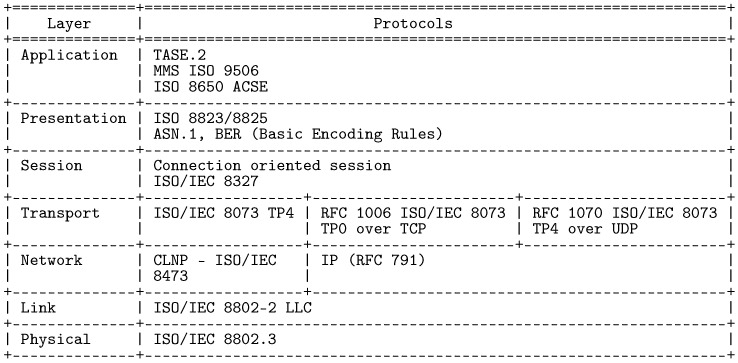
TASE.2 ISO/OSI communication model.

TASE.2 protocol builds upon a well-known ISO/OSI model for packet networks, see Figure 1. On the transport layer, transmission control protocol (TCP) ensures data transmission by default utilizing port 102. The object and function model of TASE.2 is defined on the application layer but uses additional protocols of the ISO family throughout the whole vertical model. Notably, TASE.2 heavily relies on Manufacturing Message Specification (MMS), designed to transfer real-time process data and supervisory between network nodes.

It is worth mentioning that TASE.2 by itself does not provide a direct method for authentication or encryption but instead utilizes a well-established security suite of underlying TCP/IP stack. More specifically, the transport layer security (TLS) encryption mechanisms are involved. Further, resource access is controlled by the Bilateral Tables (BLT), which represents a mutual agreement between two TASE.2 nodes. The BLT represents a set of accessible objects, variables, and services the nodes agreed on. The TASE.2 protocol defines two basic types of services:

- Operations: requests to the server initiated by the client node typically followed by a server response,
- Actions: server-initiated functions.

Other data and control elements between client and server can be exchanged as data objects. TASE.2 defines eight types of these objects, namely: (i) *Association*, (ii) *Data Value*, (iii) *Data Set*, (iv) *Transfer Set*, (v) *Account*, (vi) *Device*, (vii) *Program*, and (viii) *Event* [8,9,10,11].

Further, TASE.2 functionality is defined in multiple parts of IC 60870-6 standard as a part of *Conformance Blocks* [8,9,10,11]. In total, the TASE.2 standard nine of these blocks:

- Block 1: *Periodic Power System Data*—periodic transfer of power system data with time stamps.
- Block 2: *Extended Data Set Monitoring*—non-periodic transfer of data, such as system changes detection.
- Block 3: *Block Transfer Data*—mechanism of efficient data transfer of not structured values.
- Block 4: *Information Message*—mechanism of text or binary message transfer.
- Block 5: *Device Control*—mechanism for transferring a request to operate from another TASE.2 node.
- Block 6: *Program Control*—allows to control server site from the client node.
- Block 7: *Event Reporting*—mechanism for reporting system events.
- Block 8: *Additional User Object*—mechanism for transferring scheduling, accounting, and other information.
- Block 9: *Time Series Data*—mechanism for time series data transmission.

### 2.2. TASE.2 Join Procedure

From the perspective of communication architecture, the TASE.2 protocols two inherently different approaches for accessing requested data. These two approaches, distinguished by how the data is retrieved, are denoted as PUSH and PULL types of communication. In addition, TASE.2 represents a connection-oriented service; thus, two communicating nodes always create a client-server connection prior to the actual data transmission. In other words, one node represents a server (passive node) listening for any incoming connection from clients (active nodes). Notably, though the server can handle multiple connections, the client can be connected to only a single node at a time.

**Figure 2:**
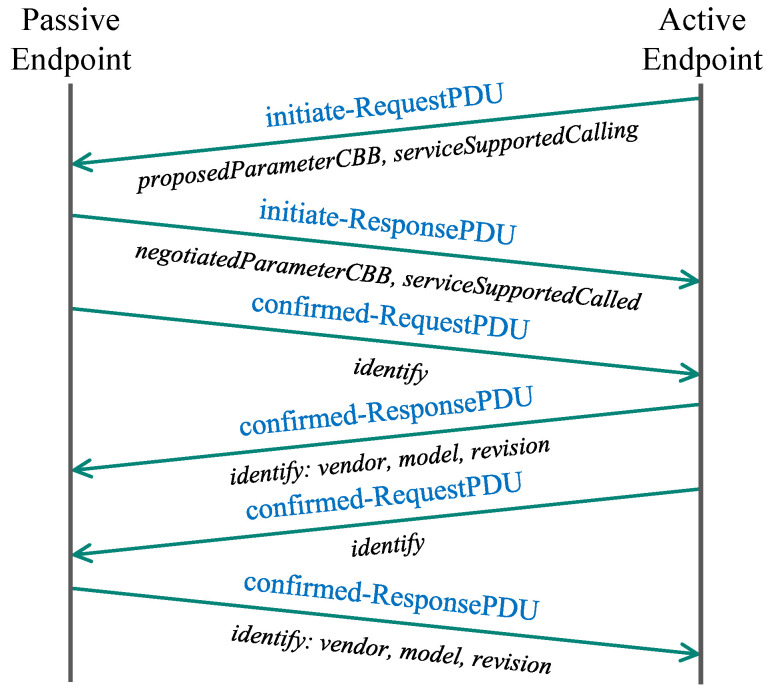
TASE.2 join procedure.

When the passive endpoint (server) binds to the socket, the connection procedure may be initiated by the active endpoint (client), which always starts the joining process. The client request from the active endpoint, among others, includes information about proposed conformance building blocks (CBB) and supported services. As a response, passive endpoint replies with negotiated parameters and supported services. Finally, the join procedure ends after successfully transmitting the identification information such as device vendor, model, and revision number. The whole message flow of the TASE.2 join procedure is depicted in Figure 2. It is worth mentioning that the encryption is handled by the underlying TLS protocol, which is entirely transparent for TASE.2 messages. For this reason, the TLS handshake is not depicted in the diagram mentioned above.

### 2.3. TASE.2 PUSH Model

In this scenario, the control center receives the values from the end-nodes in the network via data transfer, which is sent when the reported quantity value changes. The control center, in this case, acts as a passive endpoint waiting for the incoming connections from other active endpoints that report their current values. We call this a PUSH scenario since the data is actively pushed towards the TASE.2 server from the connected clients.

**Figure 3:**
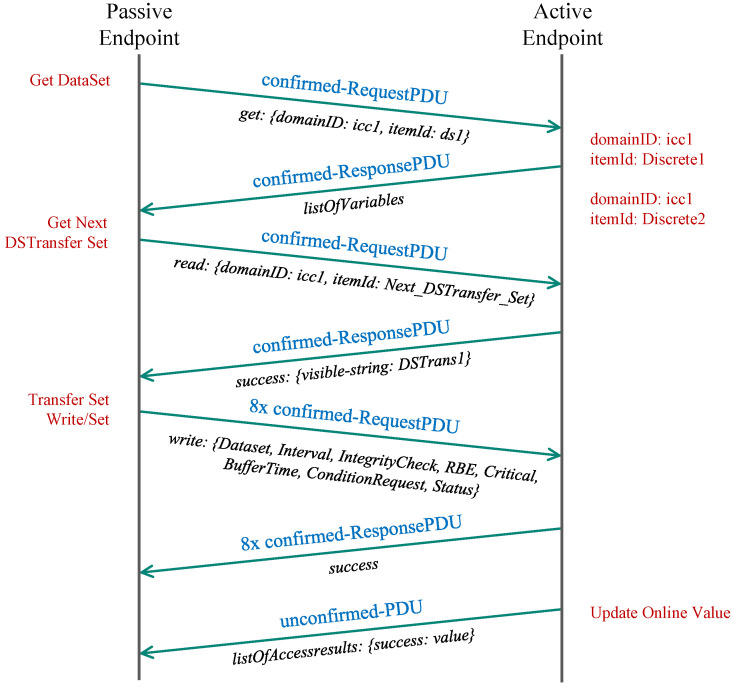
TASE.2 PUSH communication module.

First, the passive endpoint sends a confirmed request to obtain variables belonging to the selected domain (icc1) available via an appropriate data set (dts1). The passive endpoint then replies with the list of available variables. In the following step, the passive endpoint requests the next data set transfer set (DSTS), which the active endpoint acknowledges in the subsequent message. Then, the passive endpoint further negotiates the parameters of the DSTS, such as read interval, integrity check, and request conditions. Each parameter is transferred in a separate request (8 messages in our case) and is acknowledged in the same manner. From now onwards, each change of the value on the active endpoint is followed by the transmission of new DSTS containing the new value targeted for the passive endpoint. The whole communication sequence is depicted in Figure 3.

### 2.4. TASE.2 PULL Model

In this mode, the passive endpoint actively requests messages from the remote client. Thus, we refer to this scenario as the PULL model. The TASE.2 communication in this model does not utilize any DSTS, but the requested value is transferred directly in the protocol data unit (PDU) response.

**Figure 4:**
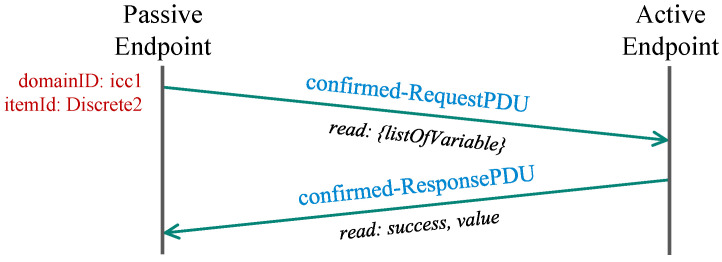
TASE.2 PULL communication module.

The passive endpoint requests the value from the remote client, which conveys the message with the appropriate status (success status in our case) and the queried value itself. The whole message flow of the PULL scenario is depicted in Figure 4.

### 2.5. TASE.2 Communication Security

As it was mentioned before, the TASE.2 security is ensured transparently by the TLS protocol [12]. Notably, both the connection initialization and the encryption negotiation are always initiated by the active endpoint (TASE.2 client). First, the active node sends the Hello message with a request to initiate authentication. Then, the server also responds with a Hello message and further sends its certificate and public key. At the same time, the server requests the client for a certificate to perform mutual verification. The provided certificates are verified against the certification authority issuing the certificates. After that, the client’s certificate, including its public key, is sent to the server that performs the same authentication procedure.

If the verification is successful, the client generates a pre-master secret (PMS) key, which is encrypted by its private key and sends it back to the server. Further, the PSM key is used to compute the master-secret (MS) key, which is used to encrypt all subsequent communication using the cipher algorithm negotiated during the connection initialization [13].

## 3. TASE.2 Generator and Collector Testbed

This chapter describes our TASE.2 testing testbed consisting of the TASE.2 traffic generator and data collection backend with load balancing functionality. The TASE.2 generator core builds upon MZ Automation ICCP/TASE.2 IEC 60870-6 library written in C language [14]. For the purposes of this research, we used its built-in JAVA application programming interface (API), which was necessary due to the use of the Apache JMeter^™^ platform for traffic generation.

The second part of the implemented testbed consists of a collecting server that is able to handle traffic from either virtualized or real TASE.2 nodes and pass the collected values to the Apache Kafka clusters through the load-balancing servers. Collected data is further stored in the high-performance No-SQL database MongoDB.

### 3.1. Developed TASE.2 Generator

To simplify the process of TASE.2 protocol testing, we implemented the proposed generator as an Apache JMeter^™^ plugin that allows setting all options via a user-friendly graphical interface with the possibility of exporting the created scenarios into the jmx file. Such an exported scenario is possible to launch on a machine without a graphical user interface. On top of that, the test case created in this way can also be part of a continuous delivery integration pipeline. Further, the use of the JMeter^™^ platform brings the additional advantage of combining multiple tests under one umbrella, which is difficult to achieve with single-purpose applications [15,16]. Therefore, integrating our generator with the already preloaded plugins makes it easy to create complex testing scenarios covering the whole communication model.

As it was already mentioned, the generator supports both types of TASE.2 communication models, i.e., PUSH and PULL. In the case of the PUSH scenario, the generator acts as an active client connecting to the passive endpoint server. Notably, to evaluate the performance of the remote server, the generator can create multiple virtual nodes on a single machine. Furthermore, each node is assigned with a unique internet protocol (IP) address; thus, the remote nodes see the generator as numerous physical machines. When the testing is finished, all assigned IP addresses are released.

For the PULL scenario, the situation is different. The generator, in this case, represents the TASE.2 server (or multiple of them) which is in the role of a passive endpoint waiting for an incoming connection. It must be noted that the created TASE.2 passive nodes must announce their presence to the active endpoints as they are not directly connected at the time of creation. For this purpose, a simple representational state transfer (REST) service is employed. This service is used to send the list of virtual stations together with the test plan duration on the generator side.

**Figure 5:**
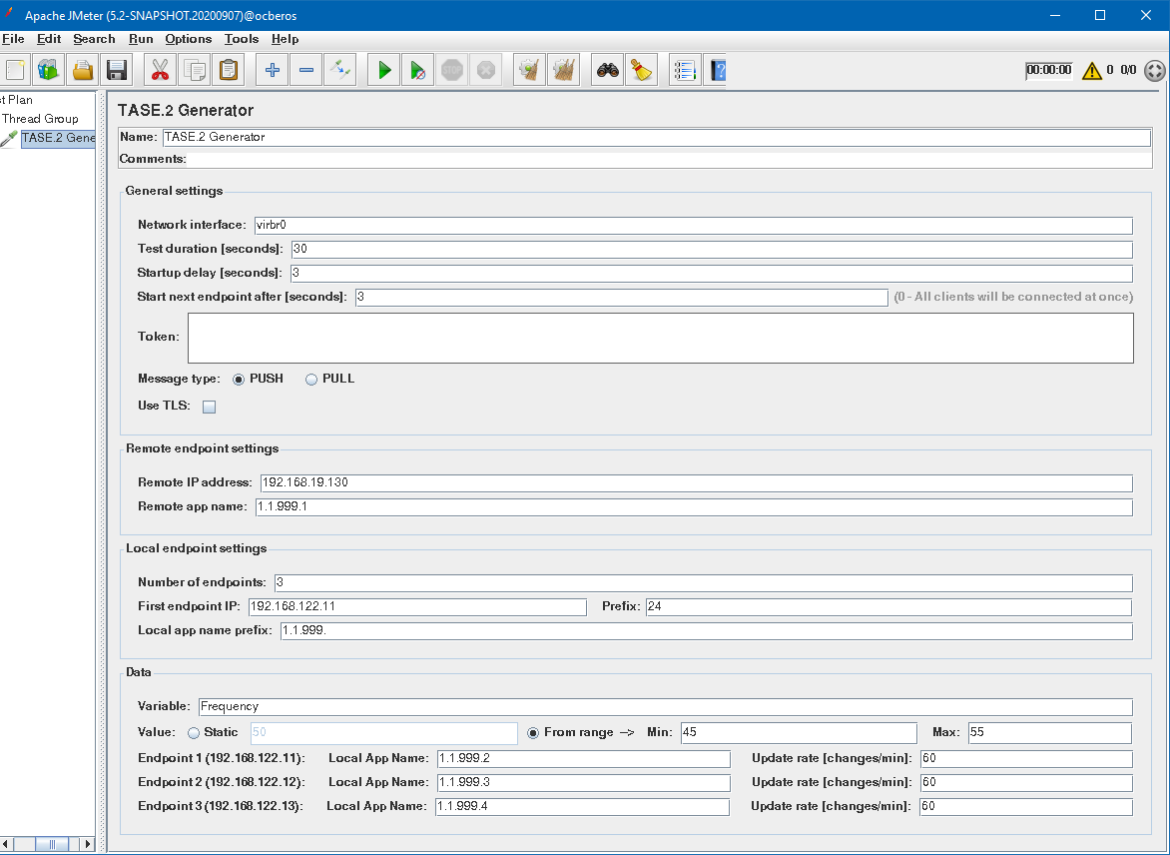
TASE.2 Apache JMeter^™^ sampler graphical interface.

As depicted in Figure 5, the generator allows users to create test scenarios with support for dynamically created nodes for both PUSH and PULL communication models. In addition, the generator further supports the staircase strategy of generating nodes when every *n* seconds, a new node is started and runs for a defined time period. When the test is finished, these nodes are terminated. The generator further supports staircase mode when the maximum number of nodes is generated in half of the simulation scenario. Then the nodes are gradually removed with the same rate *n*.

It can be seen that the configuration window is divided into three groups which are dynamically adjusted based on the selected configuration options. For example, the first group can control general settings such as scenario type, test duration, number of created nodes, and start delay. It can also be selected on which network interface the test will be run and whether TLS should be used. Moreover, if authentication is required to access the generator REST API service, the authentication token can be specified here.

The second group specifies the parameters related to the remote endpoint. It is also tightly connected to the third group representing local endpoint settings. Notably, it is essential to set the correct TASE.2 application name prefix for both local and remote nodes; otherwise, the TASE.2 communication will not work. In the last part, settings related to transmitted values are present. The reported value can be generated either statically or randomly from the specific range. Lastly, a particular application name and update interval value can be specified for each created endpoint.

### 3.2. Proposed TASE.2 Collector Backend

**Figure 6:**
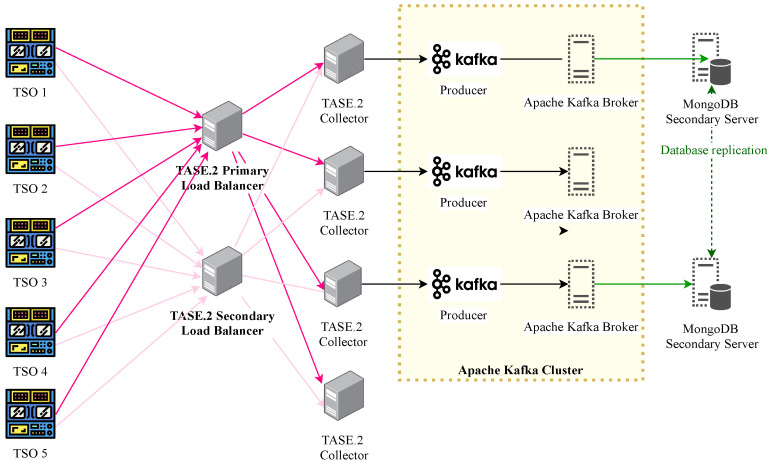
TASE.2 Collector Backend Architecture.

Due to the TASE.2 communication architecture, it is possible to establish only a direct client-server connection between an active and passive endpoint. On the other hand, our proposed solution extends the communication architecture with load-balancing nodes and Apache Kafka broker platform with high-performance database storage. These extensions allow the TASE.2 protocol to provide seamless traffic distribution and provides high data availability, as depicted in Figure 6.

Notably, high availability is achieved via redundant connection with more than one load balancer [6,17]. In our testbed, these nodes are denoted as primary and secondary load balancers. On top of that, each message which is transferred towards the TASE.2 collector cluster is marked with a unique identification and transmitted via both primary and secondary connections. Thus, the load balancers continuously track the utilization of each TASE.2 collector cluster and accordingly select the most suitable collector server for transmission. The process mentioned above represents the basic principle of traffic load balancing among the TASE.2 collector nodes, which further routes the traffic toward the Apache Kafka producer brokers.

The Apache Kafka producer then publishes all received data to the appropriate topics of the Apache Kafka cluster. This architecture allows for logical separation of real-time data ingestion from tasks that may be handled later in logical queues [18,19]. Typically, it involves further processing or storing the data in a database. It must be noted that Apache Kafka represents a fully distributed system with multiple redundant brokers and typically includes consumers and producers that can either consume or produce messages to specific topics. Hence, the high availability in the Apache Kafka platform is ensured by design [20]. Notably, with such an approach, it is clear that messages transmitted from TSOs are received redundantly (when the load-balancing topology is not broken). Hence, the broker filters these redundant messages based on their unique identifier and passes only a single copy to the following topic.

**Figure 7:**
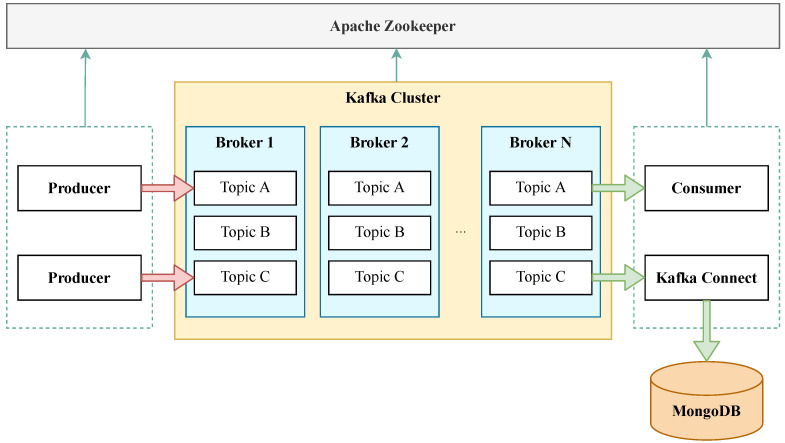
Apache Kafka Ecosystem Architecture.

Other elements in the Apache Kafka ecosystem are connectors that allow automated data writing from external data sources to Kafka broker or, conversely, storing messages from subscribed topics to the external data sources [18]. In the context of the proposed systems, long-term storage in the form of a high-performance NoSQL database plays a crucial role. These database storages may work separately, or they can be mutually mirrored. In our work, we are utilizing the second option with two MongoDB servers operating in replica set mode. Finally, the Apache Zookeeper instance manages the whole cluster, responsible for orchestrating all underlying subsystems. The overall look on the Apache Kafka platform is depicted in Figure 7. It is worth mentioning that our proposed scheme provides high availability over all verticals of the communication chains, i.e., (i) TSO to load balancer, (ii) load balancer to TASE.2 Collector, and (iii) Kafka broker to MongoDB.

### 3.3. Database Storage Architecture

The storage of received data is essential for future data analysis, performance evaluation, security assessment, or reliable long-term logging platform. The proposed solution of data storing is implemented using Kafka connect, which enables flexible, scalable, and reliable streaming of data between the Apache Kafka and other data warehouses. Notably, advanced filtering allows selecting only certain topics or specific aggregated metrics to be stored.

The main advantage of using this approach is that the TASE.2 collector server can be designed in a minimalistic way, where its main tasks, such as storing data to file systems or databases. It may require advanced queuing mechanisms that do not block the servers’ communication thread but are delegated to the database engine. The Apache Kafka robustly handles these tasks. The principle of data retention guarantees messages to be stored in the database even in the case of a short-term database server outage.

As the database platform, we selected MongoDB, which brings the advantage of real-time data storage, excellent scalability, and native high availability support. It is also possible to use indexes, which speed up later queries to the database significantly [21,22]. Moreover, MongoDB natively supports record timestamping, which is excellent for storing measured values [21]. The internal data structure consists of various JavaScript object notation (JSON) documents. An example of such a JSON document for a Smart Grid element with the frequency measurement is depicted in Listing 1.

Listing 1 **Sample JSON document of the measured record.**


{
   " _id " : Objec t Id ( "61039298 d838cf3e8ee14a88 " ) ,
   " frequency " : 54.500301361084 ,
   " timestamp " : ISODate ("2021 −05 −15T14 : 3 1 : 3 2 . 5 6 1Z" ) ,
   " source " : " i c c 4 6 " ,
   " app" : " 1 . 1 . 9 9 7 . 4 7 " ,
   " area " : "EU"
}

### 3.4. TASE.2 Testbed Security

As we are aware of the need for ensuring security in all individual blocks of the communication chain, the proposed solution provides data encryption and authentication on the highest available level. The communication between TASE.2 generator and load balancers, as well as TASE.2 collectors, is secured by the proven TLS protocol in the 1.2 version.

Apache Kafka cluster supports client authentication and authorization as well as server authentication since version 0.9. Authentication can be implemented using client TLS certificates or by utilizing the Kerberos protocol [23]. Notably, the network encryption allows Apache Kafka to transmit data over the untrusted networks but still ensuring data authenticity. The access control list (ACL) mechanism can be used to authorize clients and restrict users’ access to specific topics. The same level of security is also available in the database storage. MongoDB, aside from the conventional TLS security suite, provides access restrictions via the use of user accounts and roles.

## 4. TASE.2 Generator Performance Evaluation

To assess the TASE.2 generator functionality and performance, we conducted a series of measurements focusing on resource utilization and communication delay over large geographical areas. We divided the evaluation into two categories related to the *PUSH* and *PULL* communication model. On top of that, we also provide the initial result on measurements of the high availability and traffic load-balancing.

### 4.1. Measurement Scenarios and Setup

The communication delay between the TSOs and the control centers plays a crucial role in the SCADA system. Notably, its increased value may lead to severe damage to the transmission system or cause fatal injuries; hence, communication delay must be kept as low as possible. Moreover, the TASE.2 protocol is commonly used for electric distribution systems on an inter-state basis over large geographical distances. Our simulation scenario reflects this fact by deploying our virtualized TASE.2 endpoints utilizing Microsoft Azure cloud. In total, we used four Microsoft Azure datacenters, scattered all over the globe, namely in Europe, Brazil, South Africa, and Canada [24].

The use of the Microsoft Azure service allowed us to simplify the deployment process, as the automated orchestration script was created to install all components, prepare the environment, and launch the tests without the need for user interaction in all geographical locations. For this purpose, we used predefined Azure machines in *D4s_v3* and *B2s* configurations for TASE.2 collectors and Apache Kafka brokers with MongoDB, respectively. The exact hardware parameters of these machines are listed in Table 1 [25].

**Figure 8:**
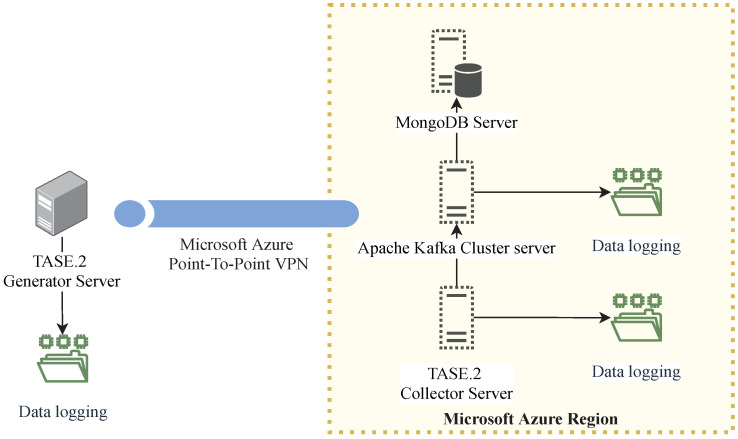
Topology of the components used during the experiments.

It must be noted that the communication delay and resource utilization measurements are primarily focused on the performance evaluation of the TASE.2 generator; hence the performance of the Apache Kafka ecosystem is not evaluated in detail, and the brokers were run on a single virtual machine. However, Apache Kafka containers would be run on different servers with multiple backups in a production environment (such an approach is utilized in our high availability and load balancing scenario). The overall look at the measurement scenario topology is depicted in Figure 8. The exact version of the used tools and software libraries are listed in Table 2.

Notably, the TASE.2 generator was situated in the data center in the Brno University of Technology network and connected to Azure regions using the Azure point-to-site virtual private network (VPN). Finally, before the actual communication delay measurements, the times on all machines had to be synchronized. For this purpose, all nodes synchronized their internal clocks against the same network time protocol (NTP) server.

### 4.2. Round-Trip-Time Delay

As the first step of our communication delay evaluation, we wanted to delineate the lower bound on the expected communication delay between the individual geographical regions. We used a well-known network tool called Packet InterNet Groper (PING) to determine these reference values, which utilizes internet control message protocol (ICMP) echo messages to obtain communication round-trip-time (RTT). The observer communication delays depicted in Table 3 were acquired by transmitting 150 B messages, having approximately the same size as the MMS protocol data unit (PDU). Notably, the depicted results are averaged from 1000 samples for each geographical location to achieve sufficient statistical confidence.

Even cursory analysis of the results confirms the basic assumptions connected with the communication distance. The lowest average delay is present at the closest node, i.e., the west Germany site. Contrary the highest average delay is between Brazil and South Africa servers. Surprisingly, in this case, the distance between these two countries is not even the longest. A slow network may cause it along the route or, more probably, the length of the communication route is not directly in line with the haversine distance. Nevertheless, the values from Table 3 should represent the lowest possible transmission delay, as they are not burdened with the overheads of higher layers.

### 4.3. TASE.2 Communication Delay

To evaluate the communication delay of the TASE.2 protocol, we used the same geographical nodes as in the previous section. However, the measurement results were not acquired from ICMP echo messages. Therefore, the delay results represent a time difference between the value update action on the client-side (active node) and the reception of the DSTS on the collector side (passive node). However, in the *PULL* scenario, the roles of active and passive endpoints are reversed. However, it is impossible to accurately evaluate communication delay due to the impossibility of ensuring precise timing of value readouts in the *PULL* scenario. The TASE.2 system only allows you to set the timestamp if the variable’s value is changed. Furthermore, since the two communication nodes can not be precisely synchronized, the delay measurements for the *PULL* communication would indicate significant jitter. Therefore, the delay measurements for the *PULL* communication model are not presented. From the logical point of view, periodical network pulling is not the optimal solution for nearly real-time communication, and the *PUSH* scenario is the preferred option.

**Figure 9:**
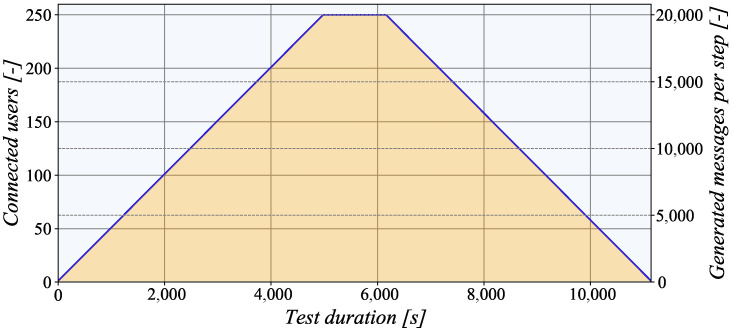
The progression of the number of messages generated in each step.

It must be noted that all experiments were performed with the TASE.2 generator that used a step testing strategy where the number of simultaneously connected endpoints was changed dynamically. In the rising phase, the generator activated a new client every 20 s, as is depicted in Figure 9. When the number of clients reached the maximum value, the generation stopped. After a certain period, the clients started disconnecting at the same rate. Notably, when the number of connected clients reached the maximum value, 20,000 messages were generated, representing a large dataset. To process such a large file, we used the open-source Pandas Python library [26].

**Figure 10:**
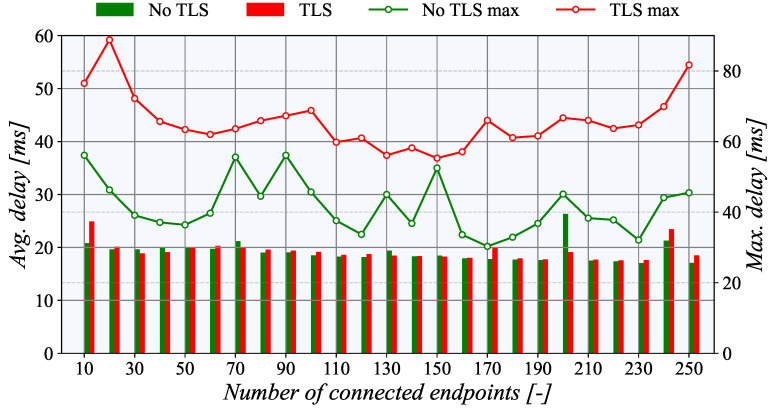
Communication delay during performed PUSH test measurements.

Figure 10 shows that the average communication delay to the nearest TASE.2 collector in Germany is below 20 ms. Enabled TLS encryption also leaves a nearly imperceptible effect on average delay; only the maximum delay increased noticeably. This behavior can be attributed to the overheads of establishing a TLS session when a new node is connected. Surprisingly, the communication delay is nearly independent of the number of active nodes. One may even claim that a higher number of connected nodes indicate lower delay. This behavior may be connected with the internal processes of the communication network as more demanding data traffics packet’s route is cached in the network elements; thus, they can go through with smaller delay.

**Figure 11:**
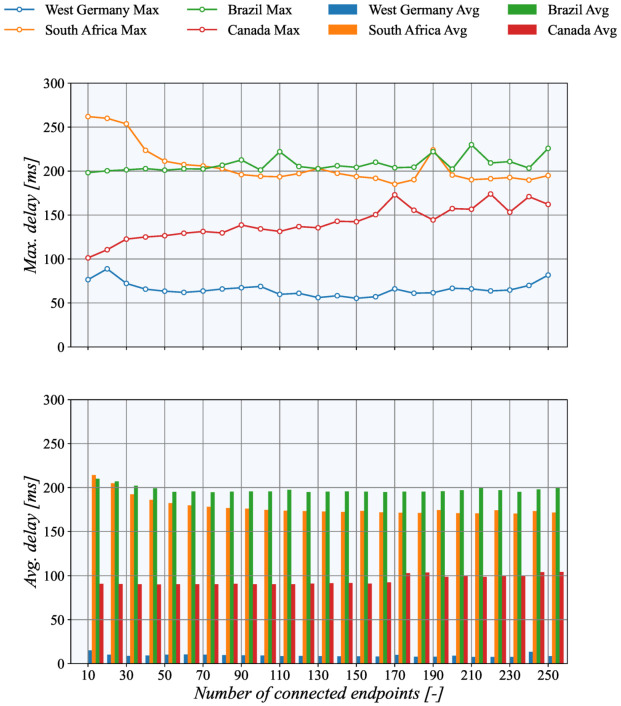
Communication delays of PUSH test measurements for different Azure sites.

The results depicted in Figure 11 demonstrate how the communication latency depends on TASE.2 collector geographical location. It can be seen that the highest delay is present when the collector is placed in the Brazil data center, while the lowest values are present for West Germany servers. These findings are in line with the data collected using the ICMP echo messages. Interestingly, the values gathered from the TASE.2 measurements are even slightly lower. It is mainly caused by the fact that the ICMP echo replies with the same 150 B message; on the other hand, TCP only sends short 40 B acknowledging messages in response. Closer inspection of the results further revealed that the West Germany node’s maximum delay is significantly higher than ICMP and averaged values. This difference may be caused by temporary network congestion during measurements.

Lastly, we also evaluated the additional delay introduced by using the Apache Kafka cluster, which was configured to store timestamps of the received records. This fact allowed us to calculate the total delay from the generation of the message until its delivery. Notably, the consumer reads the messages from the cluster remotely after 100 ms, so the Apache Kafka delay is not constant for all readings. Nevertheless, the additional Apache Kafka delay was 20 ms on average for all performer experiments, with the maximum value not larger than 78 ms. Thus, although the overall cluster delay is low due to the nature of platform operation, it is not recommended to use Kafka consumers for tasks that must be executed in real-time. Such jobs should be handled as close to the source as possible.

### 4.4. System Resources Utilization

In the case of system resource utilization, we measured the CPU and memory load of the TASE.2 generator server for both *PUSH* and *PULL* scenarios. Moreover, for the *PUSH* model, we also provide measurement results of the TASE.2 collector node, including network utilization evaluation. Notably, the CPU utilization and memory usage represent the load of the entire virtual machine provided by the operating system.

**Figure 12:**
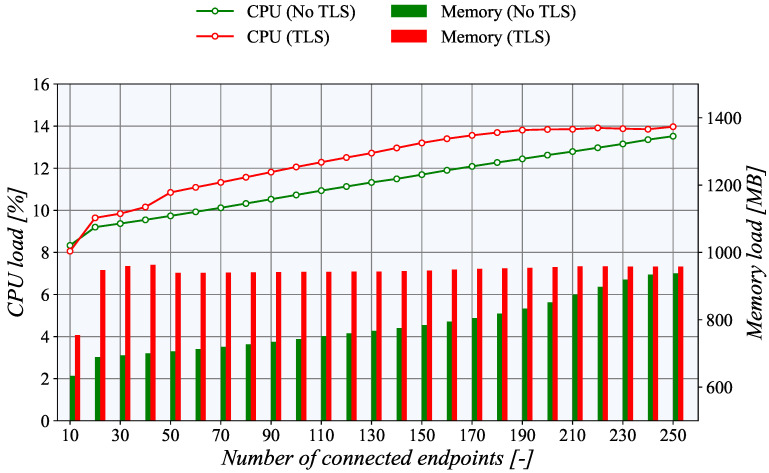
Utilization of system resources on the generator side in a PUSH scenario.

**Figure 13:**
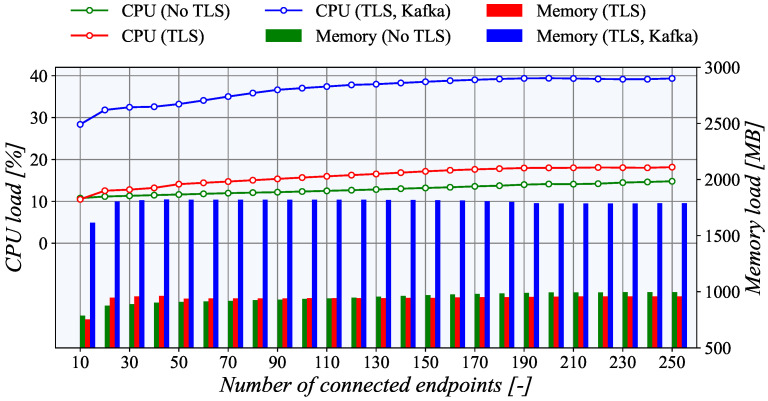
Utilization of system resources on the collector side in a PUSH scenario.

The results of the *PUSH* model depicted in Figure 12 and Figure 13 reveal the high-performance potential of the created platform, and the hardware configuration of the selected platform has plenty of headroom for more challenging scenarios.

The generator’s utilization remains below 15% on the generator side and does not overcome 20% on the collector node even with the maximum number of simultaneously connected endpoints. As can be seen, the TASE.2 collector CPU utilization rises almost linearly with the increasing number of active clients. However, when the number of clients reaches 190, the TLS CPU utilization reaches its maximum value and does not increase with the rising number of active connections. The difference between TLS and unencrypted TCP is also only marginal at a maximum of around 3%. Furthermore, without the Apache Kafka broker, the CPU utilization of the TASE.2 collector server is nearly identical to the generator side. Nevertheless, when Apache Kafka is employed, the CPU load rises significantly by almost 20%. It is a clear indication that the main bottleneck of our system is not the TASE.2 communication itself but the subsequent message processing. Overall, the described findings show that, if necessary, another instance of the generator for the *PUSH* model can be run concurrently, and the number of generated endpoints can be increased up to 500 or 750 simultaneously active clients with the current hardware.

**Figure 14:**
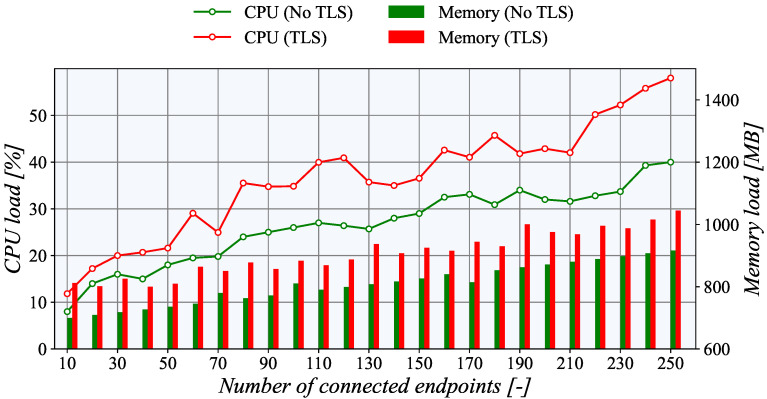
Utilization of system resources on the generator side in a PULL scenario.

Surprisingly, for the *PULL* communication model, the CPU utilization is significantly higher, as depicted in Figure 14. The steepness of the curve is more prominent. Moreover, in line with the fundamental assumptions, the CPU load with active TLS is higher. For example, with the maximum number of connected nodes, i.e., 250, the difference between TLS and unencrypted TCP is almost 15%. However, with 10 connections, the difference is only about 5%. The basic principle of data acquisition most probably causes this increased CPU load of the *PULL* scenario. In this case, the generator side creates multiple instances of passive TASE.2 nodes which have to receive and process the incoming requests for the data readouts. On the other hand, in the *PUSH* model, the updated values are transmitted to the server with each update. Basically, it means almost half of the operations compared to the *PULL* scenario.

From the perspective of memory usage, the collector side of the *PUSH* scenario indicates exciting results. When the TLS encryption is not employed, the memory usage increases linearly with the number of connected nodes. However, with TLS, the memory usage is higher but nearly constant, independently of the number of connections. This behavior is probably connected with the TLS implementation of the underlying *libtase2* library. On the TASE.2 collector side, the memory consumption is nearly constant regardless of the number of active connections. This behavior is expected as only one instance of a TASE.2 passive node is running, and the number of clients is allocated beforehand. The only surprising finding is that for more than 130 clients, the memory usage of the TLS connection is slightly lower than the unencrypted one. However, this slight discrepancy may be caused by the overall utilization of the TASE.2 collector server. However, the Apache Kafka impacts the memory the most, contributing more than 50% of the total usage. Finally, for the PULL scenario, the memory utilization rises linearly with the number of connected clients. As expected, the memory load of the TLS connection is higher by about 20%.

### 4.5. Network Utilization

**Figure 15:**
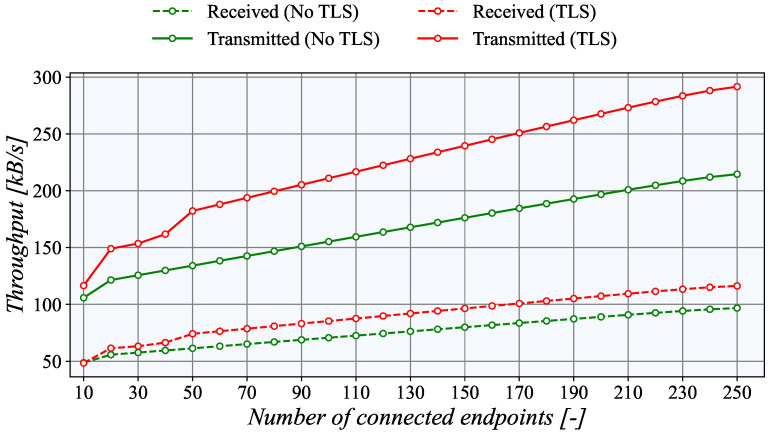
TASE.2 network interface utilization during PUSH measurements.

To provide a full assessment of the TASE.2 generator, we also present network utilization results. For this purpose, we created a dedicated network interface in the Microsoft Azure platform for TASE.2 traffic, which was connected to the other sites using Azure VPN. This interface was monitored on the generator side. As presented in Figure 15, the network utilization increases linearly with the number of active endpoints.

Notably, the TLS uses about 25% more bandwidth than the unencrypted connection due to protocol overheads. The network utilization in the uplink direction (transmitted data) is significantly higher as the actual measured values are sent in this direction. The downlink (received data) channel serves only for acknowledgments, which is less than a third of the total amount.

### 4.6. TASE.2 Load Balancing

As high availability, performance, and scalability are the crucial requirements of modern SCADA systems, we evaluated the functionality and performance of our proposed system. Our solution is designed to distribute the traffic to several TASE.2 collector servers. Unfortunately, TASE.2 protocol does not provide any form of load-balancing tools; hence in our solution, we implemented primary and secondary load balancers built upon the open-source HAProxy server [27]. Overall, the load balancer forwards the incoming traffic towards the TASE.2 collector servers designated as S1, S2, and S3.

**Figure 16:**
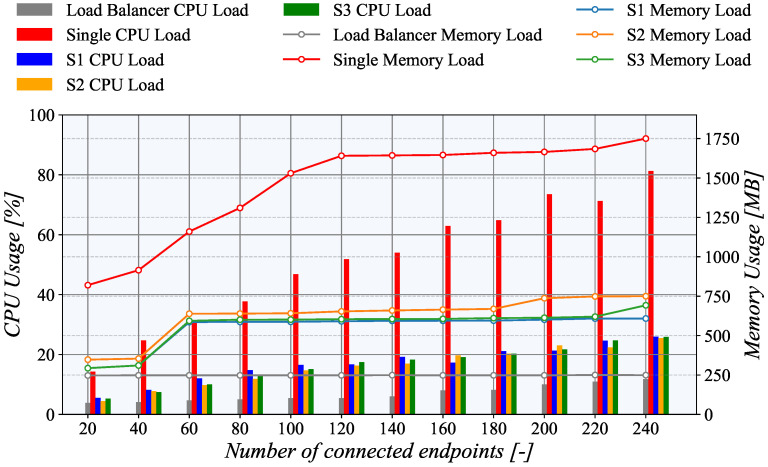
Utilization of system resources on the collector side during load balancing experiment.

To test the upper limits of our system, we modified our scenario to generate a new TASE.2 endpoint every 20 s. With the ultimate goal of increasing TASE.2 collector servers system load, each endpoint transmitted 20 messages per second. Then, when the number of active connections reached 250, the test was terminated. Notably, in maximum, the load-balancing server had to process 5000 messages every second. All tests were conducted in the Microsoft Azure ecosystem, and the collector server was downgraded to Azure B2s with dual-core CPUs for a higher clarity of results. The measurement results depicted in Figure 16 show CPU and memory usage of individual machines during the experiment. The same test was also run with a single server configuration to show the difference in resource utilization when load-balancing is not employed.

Our initial results confirm that the load balancing is working as the CPU load was evenly distributed among all servers, i.e., S1, S2, and S3. It is also clear that the CPU utilization increases linearly with the number of active connections. However, in the case of memory load, the results differ significantly. The memory usage still rises with the number of connected clients, but it is not a linear dependency. Notably, it resembles a staircase pattern as it steeply rises between 40 and 60 endpoints, and then it is nearly constant.

Finally, the load balancer CPU utilization is also linearly dependent on the number of active connections. However, during the whole testing, it was under 15%, even with 240 active nodes. Surprisingly, memory utilization is a nearly constant independent of the number of active connections. This behavior is primarily caused by the fact that the load balance only forwards the TCP connection, but further does not process any TASE.2 data.

## 5. Conclusions

In this work, we approached the problem of evaluating TASE.2 protocol performance in a multi-node SCADA environment. The main focus was given to communication delay, system resources, and network utilization assessment. Moreover, the main benefit of this paper lies in the implementation and verification of TASE.2 traffic load balancing and high availability. Together with the communication delay, these two later mentioned representing the most crucial SCADA systems requirements as unexpected system blackout or increased communication delay may have fatal consequences.

Thanks to the fully redundant topology, the presented framework allows maintaining the working communication even in the case of TASE.2 collector or communication link dropouts. Moreover, the system modularity provides unlimited horizontal scalability, allowing for creating extensive stress tests scenarios for both virtual and real environments. As the proposed system implements both generation and collection nodes, complex simulation scenarios can be designed. It allows simulating the real-world environment safely in a fully virtualized manner without the possibility of damaging the system. This follows the modern trend of the digital twins.

The designed framework consists of two main parts: (i) TASE.2 traffic generator and (ii) data collection back-end. Firstly, data generator is possible to use for performance evaluation of the stability of different TASE.2 systems from different vendors as it integrates both the PUSH model and PULL model. Secondly, the data collection back-end enables storing all the information from the TASE.2 systems and further processing.

The performance evaluation was done using the implemented traffic generator, where up to 250 active connections were established. The performed experiments were done for five geographically separated networks. The gathered data shows the high performance of the designed framework. Even without the load balancing integration, the number of active connections was possible to increase up to 700 (based on the utilized hardware platform). Once the load-balancing was integrated, the performance improved significantly. The results show the CPU usage of the load-balancer below 15% while processing 5000 messages per second. Thus, it makes it possible to achieve up to a 7-fold improvement of performance resulting in processing up to 35,000 messages per second.

## Figures and Tables

**Table 1:** Hardware equipment of used servers.

	Generator Server	Azure D4s_v3	Azure B2s
CPU	Xeon CPU E5-2670	Xeon Platinum 8272CL	baseline CPU
vCPU cores	4	4	2
System memory [GB]	8	8	4
SSD capacity [GB]	40	x	8

**Table 2:** Used software equipment.

Software Name	Software Version
Apache JMeter	5.2.1
Apache Kafka	6.2.0
HAProxy	2.0.13
ICCP/TASE.2 IEC 60870-6 Protocol Library	2.1.13
Java Development Kit	1.8.0u292
Linux Kernel	5.8.0
MongoDB	3.6.8
Ubuntu Server	20.04

**Table 3:** Measured communication RTT in ms between the sites used in the experiment.

	Czechia	West Germany	South Africa	Brazil	Canada
**Czechia**	-	11.652	192.558	202.295	109.075
**West German**	11.652	-	183.197	191.770	98.314
**South Africa**	192.558	183.197	-	353.562	248.631
**Brazil**	202.295	191.770	353.562	-	131.953
**Canada**	109.075	98.314	248.631	131.953	-

## Data Availability

Not applicable.
